# Insulin Resistance/Hyperinsulinemia Should Be Considered in the Prevention and Treatment of Essential Hypertension

**DOI:** 10.3390/biomedicines13123102

**Published:** 2025-12-16

**Authors:** Serafino Fazio, Flora Affuso

**Affiliations:** 1 School of Medicine, Federico II University, 80131 Naples, Italy; 2 Independent Researcher, 80129 Naples, Italy; flora.affuso70@gmail.com

**Keywords:** insulin resistance, hyperinsulinemia, hypertension, essential hypertension, cardiovascular disease, type 2 diabetes

## Abstract

Essential (primary) hypertension is defined as such because it does not have an identifiable cause, which is different from secondary hypertension which generally recognizes renal, endocrine, or blood vessel disorders. It is a recognized important risk factor for cardiovascular diseases and it is well known that it is largely more prevalent than secondary hypertension, accounting for 90–95% of affected adults. Therefore, in the vast majority of these cases, we do not recognize the real causes of hypertension, but we treat it without addressing the point that we should be treating hypertension by directly counteracting the cause. Essential hypertension (EH) and insulin resistance (IR) are frequently associated in the same patients, and it should be noted that EH has a comparable and rather high prevalence in adults worldwide, i.e., between 30 and 40%. Also, IR with associated hyperinsulinemia (Hyperin) is an important risk factor for cardiovascular diseases. It can cause hypertension through multiple established pathophysiological mechanisms. IR/Hyperin could be an important cause of most of the cases of essential hypertension. The aim of this Perspective article is to analyze whether the time has come to identify IR as a main contributing cause of essential hypertension. This could produce a better cure for hypertension that is also aimed at treating insulin resistance.

## 1. Introduction

Despite improved preventive and therapeutic approaches to cardiovascular diseases in recent decades, they are still the leading cause of death [1]. So, it is necessary to make greater efforts to lower the prevalence of cardiovascular diseases, reducing their mortality. There are numerous risk factors for cardiovascular disease but, among these, high blood pressure is a risk factor that has been recognized for many years and is very widespread in the world adult population, increasing the occurrence of pathologies such as stroke, coronary artery disease, heart failure, atrial fibrillation, and renal failure. High blood pressure is an important cause of damage to the vascular system and the heart, leading to serious health problems, resulting in an increase in hospitalizations and deaths, with a significant burden in healthcare spending [2,3]. High blood pressure is classified in two main forms: essential hypertension (EH, also known as primary hypertension), which does not have an identifiable cause, and secondary hypertension, in which kidney, endocrine, or blood vessel disorders may be recognized [4,5]. In the past, the adjective “essential” was meant as a necessary mechanism of hypertension to ensure good organ perfusion. Nowadays, any form of hypertension is unequivocally recognized as an important risk factor for cardiovascular diseases [6]. EH is significantly more prevalent than secondary hypertension, accounting for about 90–95% of adult cases [2,7]. Furthermore, the prevalence of EH has been shown to be progressively increasing over the last few decades, at the same time as insulin resistance (IR) [8,9,10]. IR is a pathological metabolic condition caused mainly by an unhealthy lifestyle, but in some cases also by genetic factors. It is well known that this condition precedes type 2 diabetes mellitus by many years and is its main cause. Given the high prevalence of EH compared with secondary hypertension, in the vast majority of cases we do not know the cause of hypertension and, therefore, we treat it without going directly to the point, that is, treating hypertension by directly counteracting its cause. We should ask ourselves “Is it possible that in 90–95% of cases of hypertension the causes cannot be identified?” Recognizing the causes of hypertension would certainly allow for more appropriate therapy. The aim of the present Perspective article is to verify and suggest whether IR with associated hyperinsulinemia (Hyperin) can be considered an important contributory cause in the development and progression of EH.

## 2. The Point

“Is it really true that we ever do not know what causes essential hypertension? Most likely not”. In fact, if we analyze this problem in more depth, we must certainly take into serious consideration the fact that EH and IR are strictly associated in most patients [11,12,13,14,15]. Insulin resistance is a very frequent condition in which the resulting effect on the carbohydrate metabolism of a given amount of insulin is inadequate for glucose homeostasis. For this reason, in order to maintain blood glucose levels within the normal range, the pancreas must secrete, as long as it can, significantly higher amounts of insulin [9,10]. This produces increased circulating insulin levels, namely hyperinsulinemia, which is a constant and essential feature of IR [9,10]. This chronic overproduction of insulin, in addition to having the beneficial purpose of maintaining blood sugar levels within the normal range also has, over time, a harmful effect on organs and vessels, leading to severe complications. Once the pancreas is exhausted and the amount of insulin secreted becomes insufficient, overt type 2 diabetes occurs. It should be noted that EH and IR have a comparable and rather high prevalence in adults in developed and developing countries, i.e., between 30 and 40% [2,9,10,16,17], and both are expected to progressively increase in prevalence worldwide in the coming years. The association between insulin resistance and hypertension is well established across large-scale population studies. Four meta-analyses involving more than 400,000 participants confirmed a strong relationship between insulin resistance and the risk of developing hypertension. Specifically, individuals with higher insulin resistance metabolic score (METS-IR = ln[(2 × FPG mg/dL) + TG mg/dL] × BMI kg/m^2^/ln[HDLc mg/dL]) values had a 1.67-fold increased risk (95% CI:1.53–1.83) of developing hypertension, while those with elevated HOMA-IR showed a 1.43-fold increased risk (95% CI: 1.27–1.62) and those with high fasting insulin levels had a 1.54-fold higher risk (95% CI: 1.34–1.76) [17,18]. Long-term cohort data further support these findings. In the 20-year CARDIA study, participants in the highest insulin quartile had an 85% greater risk of developing hypertension (1.85; 95% CI: 1.42–2.40) compared with those in the lowest quartile [19]. A Mendelian randomization study provides support for a strict relationship between genetically determined IR and an increased risk of hypertension and confirms that insulin resistance may directly contribute to blood pressure elevation, even after accounting for confounding metabolic factors [20]. Furthermore, physiological studies performed with a euglycemic insulin clamp have demonstrated that EH per se is a state of IR. Indeed, an average reduction of approximately 40% in the ability of a physiological Hyperin to stimulate body glucose uptake has been highlighted in lean, non-diabetic subjects with untreated EH [15,21]. In another study performed by evaluating insulin sensitivity with the euglycemic clamp in 1073 European adults, aged between 30 and 60 years and followed subsequently for 3 years, it was demonstrated that systolic blood pressure (SBP) at the baseline was higher in more insulin-resistant women. After adjusting for age, BMI, baseline SBP, and other covariates, low insulin sensitivity predicted a longitudinal rise in SBP in women but not in men. However, normal or elevated insulin sensitivity was associated in all patients with a lower rise in SBP over time [22]. Insulin is a hormone, whose hematic concentrations are essential for healthy homeostasis, exactly as any other hormone, such that its levels being chronically low or high are at the base of many health issues. In medicine, all hormonal abnormalities are treated in an attempt to normalize hormone levels. Indeed, it is well known that we treat, as soon as we become aware of it, hypercortisolism, known as Cushing’s disease, an excess of growth hormone that produces acromegaly, a high circulating thyroid hormone that produces hyperthyroidism and so on, all important pathologies that cause rather evident symptoms and, often, somatic changes. Instead, at least until today, IR with associated Hyperin is treated only when prediabetes or overt diabetes are already present. Hyperinsulinemia is treated only if its cause is an insulinoma, which is a rare tumor of the pancreas that releases excess insulin, causing very symptomatic episodes of hypoglycemia [23]. On the contrary, Hyperin associated with insulin resistance is paucisymptomatic, even if it causes major damage in the medium/long term. There is extensive scientific literature, accumulated over the years, showing that Hyperin associated with IR causes progressive kidney impairment, liver steatosis, heart, and vascular system damage [24,25,26,27,28,29], but, nevertheless, for this form of Hyperin there are not indications for treatment in the guidelines. The silent and dangerous action of Hyperin represents an important health issue as already largely discussed elsewhere, and it could be considered a silent killer, since it produces many important alterations to the human organism and has been found to be associated with an increased risk of mortality [30,31,32]. Returning to the purpose of this article, it is necessary to underline the multiple pathophysiological mechanisms at the base of arterial hypertension during IR. Hyperinsulinemia, which is characteristic of IR, can cause hypertension by different mechanisms: 1. it causes an alteration in circulatory homeostasis, as it leads to the increased secretion of endothelin-1 (ET-1) at the vascular level and, conversely, a reduction in the secretion of nitric oxide (NO), resulting in endothelial dysfunction and vasoconstriction [29,30,31]; 2. it also causes an increase in the secretion of norepinephrine, another hormone with vasoconstrictive action [33,34]; 3. excess insulin, interacting with its receptors in the renal tubules, causes the increased reabsorption of sodium and water, leading to an expansion of blood volume [35,36]; 4. advanced glycation end products are increased in insulin resistance and are related to a rise in vascular stiffness in hypertensive patients [37]; 5. insulin resistance with associated Hyperin can directly stimulate components of the RAAS [38,39]. All these mechanisms, even if we cannot know their relative contribution, which can vary from person to person, to hypertension development, taken together can easily explain the onset of arterial hypertension in individuals with IR. Nevertheless, although it is clearly demonstrated that IR is a key component of arterial hypertension, still today it does not have a nosological identity, and yet we classify these forms of hypertension as essential (primary), i.e., as forms of hypertension of unknown cause, while in most cases there could be the important contributory cause of IR/Hyperin. On the other hand, hypertension itself can, in turn, worsen insulin sensitivity through vascular stiffness and neurohormonal activation [40]. Hyperinsulinemia associated with IR, in addition to being a main cause of arterial hypertension, also produces relevant direct damage to the cardiovascular system due to its mechanisms as a growth factor. In fact, hyperinsulinemia associated with the condition of insulin resistance hyperstimulates the Mitogen-Activated Protein Kinase/Extracellular Signal-Regulated Kinase (MAPK/ERK) pathway, not only promoting ET-1 release, but insulin also directly promotes cell growth by binding to the Type I Insulin-Like Growth Factor (IGF) receptor and activating phosphorylation pathways within the cell, including those involving ribosomal proteins; in addition, insulin influences the liver to produce and release IGFs, primarily IGF-1, which are potent mitogens (substances that stimulate cell division) [41,42]. Therefore, even if we can perfectly control blood pressure with antihypertensive drugs, we do not completely eliminate the risks associated with hyperinsulinemia. Although the gold standard for diagnosing insulin resistance is the euglycemic hyper insulinemic clamp, this test may not be used for screening purposes as it is too laborious, expensive, and invasive, so it is only used for research purposes [10,43]. The diagnosis of insulin resistance in patients with essential hypertension is based on a combination of clinical evaluation and simple laboratory tests, including fasting blood glucose, glycated hemoglobin (HbA1c), the homeostasis model assessment for insulin resistance (HOMA-IR) index, and the triglycerides–glucose (TyG) index. Insulin resistance can manifest itself with nonspecific symptoms such as weight gain (especially abdominal), fatigue, difficulty concentrating, increased appetite, and an altered lipid profile (high triglycerides and low HDL cholesterol) [9,10]. The pathophysiological recognition of insulin resistance as an important, if not the sole, cause of essential hypertension should pave the way for a targeted approach to this disease, from the point of view of both prevention and treatment. Treating insulin resistance not only may counteract the pathological mechanisms discussed above but also should optimize medical antihypertensive treatments currently in use, leading to a better control of arterial hypertension, using lower doses of antihypertensive medications. An intervention against insulin resistance, working synergically with antihypertensive drugs, may reduce side effects and act better on the cardiovascular risk reduction effect, preventing or at least slowing down the development of overt diabetes, that represent the final step of years of insulin resistance. Once more the point is to counteract the underlying causes of a disease and not to treat its clinical manifestations.

The prevalence of IR in patients with EH has been increasing over the years, from 25 to 30% in 1993–1995 studies to 50% in a 2009 study [43,44,45]. To address the issue of the growing prevalence of EH, we must therefore tackle the problem of preventing the development of IR, which is rapidly increasing, worldwide, in the general population and also in children, due to the progressive worsening of lifestyle in a large percentage of the world’s population. It is well known that hypertension and IR are strictly associated in subjects with overweight, obesity (particularly central obesity), and metabolic syndrome, whose prevalence is rapidly increasing worldwide, especially in developed and developing countries [44] as a result of unhealthy lifestyles. It is necessary to teach and explain that, where present, unhealthy lifestyles must be changed, as they are too often associated with an increase in caloric intake, particularly through the excessive consumption of carbohydrates and ultra-processed foods, and a reduction in daily physical activity, if not outright sedentary lifestyles [9,17,44]. In conditions of IR, the daily intake of increased quantities of carbohydrates causes significant increases in insulin secretion by the pancreas at meals, leading to further dangerous increases in circulating insulin levels. Lifestyle interventions, like a reduction in caloric intake mainly by reducing sugars and refined carbohydrates, combined with a constant increase in physical activity, can succeed in reversing this trend. Although lifestyle modification remains a key component of hypertension management in the presence of insulin resistance, when insulin resistance is confirmed and this type of intervention is not sufficient, insulin-sensitizing substances should be used in association with common antihypertensive pharmacologic treatments, such as RAAS blockade with ACE inhibitors or angiotensin receptor blockers. Many substances, both natural and pharmaceutical, with insulin-sensitizing effects are available. Among the drugs those that we consider to be supported by good and sufficient scientific literature are metformin, sodium-glucose transporter 2 inhibitors, and glucagon peptide-1 receptor agonists [45,46,47]; among natural substances, there are berberine, L-arginine, quercetin, silymarin, pyrroloquinoline quinone, and mormodica charantia [48,49]. We will not go into detail about the mechanisms and efficacy of these substances because this has already been investigated extensively elsewhere [26,27,28,49] and because this is not the purpose of this perspective article; however, all have a demonstrated ability to counteract insulin resistance and to decrease circulating insulin levels. In addition, many of these substances are also supported by the scientific literature, showing that their use can reduce cardiovascular risk [50,51,52,53,54,55,56,57,58,59,60,61,62,63]. It has been shown that many sensitizing insulin substances, in addition to having an effect in reducing insulinemia, can also have a beneficial effect on blood pressure control [64,65,66,67,68,69,70]. For example, a study, carried out in 140 subjects with arterial hypertension without diabetes but with Hyperin, compared the effects of metformin 500 mg three times a day (68 pts) with those of fosinopril 10 mg per day (72 pts). Treatments were randomized and, if the target blood pressure value (<140/90 mmHg) was not reached after 4 weeks of therapy, a combined treatment of metformin plus fosinopril was performed in these patients for another 4 weeks. Insulin levels were also measured fasting and after 30 and 120 min after oral glucose loading curve basally and after 4 and 8 weeks of treatment in the different groups. The results showed a comparable antihypertensive effect of metformin and fosinopril after the first 4 weeks of treatment. The combination resulted, after another 4 weeks of treatment, in further blood pressure control in patients who had not reached the blood pressure target with the two substances separately. Fasting insulinemia, and those at 30 and 120 min after the loading curve, were significantly reduced by metformin after 4 and 8 weeks [64].

**Figure 1:**
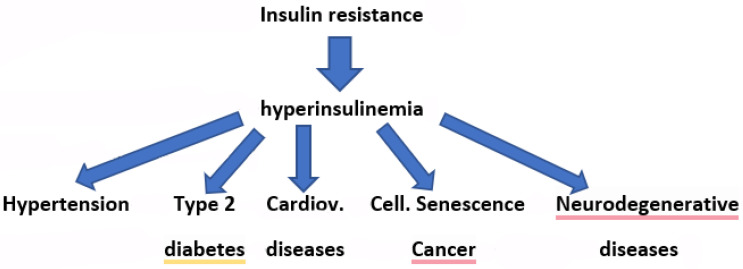
Insulin resistance and associated hyperinsulinemia is a cause of type 2 diabetes, and is a risk factor for cardiovascular diseases, cellular senescence and cancer, and neurodegenerative diseases. Cardiov.: Cardiovascular; Cell.: Cellular.

It is also necessary to underline that IR with associated Hyperin, in addition to being strictly associated with EH, is also a cause of type 2 diabetes, the development of cardiovascular diseases, cellular senescence and tumors, and is a risk factor for neurodegenerative diseases [28,29,71,72,73,74,75,76,77] (Figure 1).

We have been working on this topic for many years and we have come to the conclusion that it should be necessary that the scientific community and national health institutions conduct definitive studies into the issue of IR/Hyperin to verify with absolute certainty whether this condition should be considered a determining cause of major diseases such as hypertension, type 2 diabetes, and so on, because if this were the case, it would be important to promote the early screening of this pathological condition in the general population in order to start a prompt preventive strategy or therapeutic treatments to protect public health and reduce the burden of healthcare costs [78].

## 3. Conclusions

Most forms of EH are strictly associated with IR, but there is not yet sufficient scientific evidence to demonstrate a causal link between IR with associated Hyperin and EH. Essential hypertension, or primary hypertension of unknown cause, is treated with medications that are fairly effective in controlling it, but they do not directly address the underlying cause which often results in hypertension not being adequately controlled and not eliminating the risks directly linked to the cause of hypertension. In the cases of hypertension associated with IR/Hyperin, probably, a combination treatment of hypertensive drugs with insulin-sensitizing drugs could improve blood pressure control by reducing insulin levels which in themselves can be a cardiovascular risk factor [64]. Furthermore, like all chronic medications, antihypertensives can produce side effects, resulting in poor patient compliance. Insulin resistance with associated hyperinsulinemia should be studied to verify whether it is one relevant contributing cause of essential hypertension. This would lead to a better preventive and therapeutic approach, reducing its prevalence and improving its control, with better prognostic results and reduced healthcare costs.

This review has many limitations because there are many confounding factors between IR/Hyperin and EH, such as overweight and obesity, and population heterogeneity, and, unfortunately, we only have studies with a limited number of cases that demonstrate the beneficial effects on blood pressure of insulin-sensitizing substances. Furthermore, we do not have sufficient scientific literature showing a causal link between IR/Hyperin and EH.
